# P‐glycoprotein modulates the fluidity gradient of the plasma membrane of multidrug resistant CHO cells

**DOI:** 10.1002/1873-3468.70083

**Published:** 2025-05-31

**Authors:** Roger Busche, John R. Riordan, Burkhard Tümmler

**Affiliations:** ^1^ Institute for Biophysical Chemistry and Structural Biochemistry, Fritz‐Hartmann‐Centre for Medical Research Hannover Medical School Germany; ^2^ Department of Biochemistry and Biophysics and Cystic Fibrosis Center University of North Carolina‐Chapel Hill NC USA; ^3^ Department of Pediatric Pneumology, Allergology and Neonatology Hannover Medical School Germany; ^4^ Biomedical Research in Endstage and Obstructive Lung Disease (BREATH) German Center for Lung Research Hannover Germany

**Keywords:** ABC transporter, ABCB1, membrane fluidity, multidrug resistance, *n*‐(9‐anthroyloxy) fatty acid, P‐glycoprotein

## Abstract

Cryo‐electron microscopy has yielded high‐resolution structural data of the multidrug efflux transporter P‐glycoprotein (ABCB1), but its direct and indirect interactions within the native membrane environment have remained largely unexplored. Here, we compared the fluidity gradients of plasma membranes of the drug‐sensitive CHO cell line AuxB1 and its P‐glycoprotein overexpressing derivative B30 by fluorescence anisotropy of embedded *n*‐(9‐anthroyloxy) fatty acid probes (*n* = 2, 7, 9, 12, 16) in the temperature range of 10–50 °C. The shape of the temperature profiles of probe mobility was comparable in AuxB1 and B30 membranes, but did not match. Overexpression of P‐glycoprotein smoothened the transversal gradient of the out‐of‐plane mode of rotation of the probes, which may facilitate the partitioning of hydrophobic drugs into the membrane and thereby increase the speed of P‐glycoprotein to pump the drug out of the cell.

## Graphical abstract

To explore the impact of the overexpression of the multidrug‐transporter P‐glycoprotein (ABCB1) on membrane fluidity, we compared the transversal gradient of mobility and microviscosity in plasma membranes of drug‐sensitive Chinese hamster ovary cells (AuxB1) and their multidrug‐resistant derivatives (B30) using the fluorescent *n*‐(9‐anthroyloxy) fatty acid probes (*n* = 2, 7, 9, 12, 16) as molecular rulers.

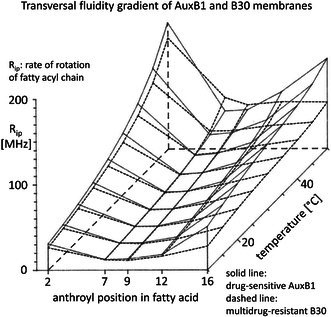

## Abbreviations

**12‐AS**, 12‐(9‐anthroyloxy)stearic acid

**16‐AP**, 16‐(9‐anthroyloxy)palmitic acid

**2‐AS**, 2‐(9‐anthroyloxy)stearic acid

**7‐AS**, 7‐(9‐anthroyloxy)stearic acid

**9‐AS**, 9‐(9‐anthroyloxy)stearic acid

**ABC**, ATP‐binding cassette

**ABCB1**, ABC subfamily B member 1

**CHO**, Chinese hamster ovary

**cryo‐EM**, cryo‐electron microscopy

**DPPC**, dipalmitoylglycerophosphocholine

**NBD**, nucleotide‐binding domain

**TM**, transmembrane domain

The ATP‐binding cassette (ABC) transporter ABCB1 (UniProt no P08183) initially named P‐glycoprotein [1] translocates drugs and phospholipids across the membrane [2, 3, 4]. P‐glycoprotein is a polyspecific multidrug transporter involved in the clearance of xenobiotics in mammals and is implicated in cancer resistance to chemotherapy [3].

P‐glycoprotein is a Type I subfamily B ABC transporter [5] with each transmembrane domain (TM) comprising six transmembrane helices and followed by a cytosolic nucleotide‐binding domain (NBD) [4]. Cryo‐electron microscopy (cryo‐EM) of the reconstituted isolated protein revealed that P‐glycoprotein forms different conformations associated with the presence or absence of ligand and/or ATP [4, 6, 7, 8, 9, 10]. The most recently published cryo‐EM study [4] could discern P‐glycoprotein in the unbound (apo) state, P‐glycoprotein in the presence of ATP/Mg^2+^, and P‐glycoprotein in the substrate‐bound and inhibitor‐bound states, respectively. According to these data, the conformation of P‐glycoprotein in the absence of substrate and ATP (apo‐ABCB1) exhibits an inward‐facing closed state with closely spaced NBDs and a closed TMD pathway central [4]. Upon substrate binding, P‐glycoprotein shifts to an inward‐facing open state with greater NBD separation that facilitates ATP binding and subsequent extrusion of substrate through an outward‐facing open conformation. Conversely, upon inhibitor binding, P‐glycoprotein shifts to an inward‐facing occluded conformation, thereby inhibiting the transport cycle [4].

The cryo‐electron microscopy studies yielded high‐resolution structural data of P‐glycoprotein in a defined lipid environment; however, the interplay between the membrane environment and the conformations of P‐glycoprotein has only recently become an emerging issue in structural biology and molecular dynamics simulation [4, 11]. Previous biochemical and biophysical studies have demonstrated that the membrane lipid composition modulates the function of P‐glycoprotein, including ATP hydrolysis, drug binding, and drug transport (summarized in refs. [12, 13]). On the other hand, overexpression of P‐glycoprotein should affect the properties of the plasma membrane. For example, P‐glycoprotein accounts for about 20% of the protein content of the plasma membrane of the multidrug‐resistant CHO cell line CH^R^B30 [14, 15]. Early work on the partitioning of the fluorescent probe merocyanine 540 in the CH^R^B30 plasma membrane suggested that the molecular packing of lipids in the outer leaflet increases with higher levels of P‐glycoprotein [16]. Likewise, when P‐glycoprotein purified from CH^R^B30 cells was incorporated into liposomes, it modulated the partitioning of fluorescent lipid analogues [17].

Since its discovery in multidrug‐resistant CHO cells [1], the features of P‐glycoprotein have been extensively studied during the last decades. However, our knowledge of the impact of the overexpression of P‐glycoprotein on the physical state of the plasma membrane is still limited [13]. To address this issue, we chose the drug‐sensitive CHO cell line AuxB1 and its multidrug‐resistant derivative CH^R^B30, because AuxB1 and CH^R^B30 share the same protein and lipid composition in the plasma membrane [1, 14, 18, 19], the different amount of P‐glycoprotein being their single distinction. Applying a set of fluorescent *n*‐(9‐anthroyloxy) fatty acid probes as molecular rulers [20], we compared the microenvironment of AuxB1 and CH^R^B30 membranes at a graded series of depths from the surface to the center of the bilayer. The in‐plane and out‐of‐plane rotations of the anthroyl ring were taken as the membrane ‘fluidity’ parameter [21]. The steady‐state fluorescence anisotropy measurements showed that the overexpression of P‐glycoprotein smoothened the transversal gradient of ‘membrane fluidity’ in a broad temperature range from 10 to 50 °C.

## Materials and methods

### Chemicals

Dipalmitoylglycerophosphocholine (DPPC) was obtained at purissimum grade from Fluka (Buchs, Switzerland). Quinine hemisulfate salt monohydrate was obtained from Sigma‐Aldrich (St. Louis, MO, USA). *n*‐(9‐Anthroyloxy) fatty acids (*n* = 2, 7, 9, 12, 16) were purchased from PL Biochemical's (now Thermo Fisher Scientific, Waltham, MA, USA) (2‐AS, 2‐(9‐anthroyloxy)stearic acid; 7‐AS, 7‐(9‐anthroyloxy)stearic acid; 9‐AS, 9‐(9‐anthroyloxy)stearic acid; 12‐AS, 12‐(9‐anthroyloxy)stearic acid; 16‐AP, 16‐(9‐anthroyloxy)palmitic acid). Their purity was controlled by thin‐layer chromatography with ethanol–water (95 : 5, v/v) as solvent and absorption spectrophotometry. The individual stock solutions of the five dyes differed from their mean extinction coefficient by up to 1.2% in the range from 320 to 380 nm of the absorption spectrum. All other purchased reagents used for fluorescence measurements were of analytical grade and free from fluorescent impurities.

### Cell culture

The highly colchicine‐resistant mutant CHO line CH^R^B30 [14, 22] was selected from the glycine‐, adenosine‐, and thymidine‐requiring auxotroph AuxB1 [23] by clonal selection, yielding cell line CH^R^B3, followed by stepwise selection, yielding the intermediate cell line CH^R^C5, and finally by continuous culture in increasing colchicine concentrations, giving CH^R^B30. Aliquots of the stock of AuxB1 and CH^R^B30 cells stored in nitrogen tanks were the starting material for cell culturing.

During the study period, the cell lines AuxB1 and CH^R^B30 were authenticated by line‐specific features of *mdr1* gene copy number and expression:

- Expression profiling: MDR1 RNA transcript levels were quantified by a kinetic polymerase chain reaction assay with ^32^P‐labeled nucleotide optimized in‐house to determine hamster mdr1 RNA levels within the range of 0.001–1000 amol specific mRNA.
- Karyotyping: G‐banding [22].
- *In situ* hybridization with a *mdr1* probe to detect the homogeneously staining region of amplification of the hamster P‐glycoprotein gene *mdr1* on chromosome Z4 of CH^R^B30 as the characteristic chromosomal alteration between the parental line AuxB1 and its multidrug‐resistant derivative CH^R^B30 [22].
- Coomassie blue stain of Fairbanks gel‐separated plasma membrane proteins [1, 14, 19].

Cells were grown in α‐minimal essential medium with nucleosides (Gibco, Paisley, UK) supplemented with 10% fetal calf serum (Flow Laboratories; Gibco) and in addition for CH^R^B30 cells only with 30 μg·mL^−1^ colchicine. Cells were cultured in monolayers at 37 °C in humidified air containing 5 vol.% CO_2_. Cultures were propagated twice a week by tryptic splitting (CH^R^B30 1 : 10, AuxB1 1 : 40) for up to 1 month. Cultures were regularly checked for contamination with mycoplasma. No mycoplasma had been detected in any of the processed cell cultures.

### Membrane isolation

Washed trypsinized cells at a concentration of 5 × 10^7^·mL^−1^ in phosphate‐buffered saline were disrupted in a pump at a pressure of 1.2 × 10^6^ Pa for CH^R^B30 and 2.4 × 10^6^ Pa for AuxB1, which minimized the breakage of intracellular organelles [1]. The ruptured cell suspension was subjected to the following differential centrifugation steps: nuclear spin, 300 ***g*** for 30 min; mitochondrial spin, 4000 ***g*** for 10 min; microsomal spin, 35 000 ***g*** for 30 min. The microsomal pellet was resuspended in phosphate‐buffered saline and applied to a discontinuous sucrose gradient consisting of 10 mL 60% (mass/vol.), 14 mL of 45%, 24 mL of 31%, and 12 mL of 16% sucrose. Centrifugation was performed in an SW25.2 rotor at 76 900 ***g*** for 18 h. The fraction of plasma membrane vesicles banding at the 16/31% interface was removed, washed with 5 mm Tris/HCl, pH 7.5, and resuspended at 5–10 mg protein·mL^−1^ in the same buffer containing 0.25 M sucrose. After an aliquot had been taken for the determination of protein content (fluorometric procedure according to Böhlen *et al*. [24]), the vesicle preparation was purged with nitrogen and stored at −70 °C until used. The plasma membrane preparations contained 0.61–0.73 μmol total phospholipid per mg protein and 0.44–0.50 μmol cholesterol per mg protein [18]. To check the gross protein composition of the plasma membrane preparations that represent less than one‐half of 1% of the total cell protein [1], samples were separated by continuous PAGE with 1% (w/v) SDS and 6 M urea using the procedure of Fairbanks *et al*. [25] modified by Debenham *et al*. [26]. P‐glycoprotein made up about 20% of protein in CH^R^B30 membranes, but was not detectable by Coomassie stain in AuxB1 membranes.

### Phospholipid vesicle preparation

Unilamellar DPPC vesicles were prepared by injection according to Parce *et al*. [27]. Two mg DPPC were dissolved in 5 mL dimethylether/methanol (3 : 1, by vol.). Aliquots of 0.1 mL were injected within 30 s into 5 mL 0.1 mm potassium phosphate, pH 7, at 60 °C. Vesicle preparations were maintained at 55 °C until used.

### Fluorescence measurements

Steady‐state fluorescence spectra and emission anisotropies were measured with a RRS 1000 (Schoeffel, Westwood, NJ, USA) spectrofluorometer equipped with two grating excitation monochromators, a single grating emission monochromator, and Glan Thompson polarizers. The output voltage signal of the fluorometer was digitized and imported into a laptop for subsequent data analysis.

We followed a standardized sequence of actions. Starting in the evening after 6 pm, the absorption spectrum of 0.14 mm *n*‐(9‐anthroyloxy) fatty acid in tetrahydrofuran was recorded at room temperature. Next, the thermostated cuvette holder of the fluorometer was connected with a water bath set to 10 °C and a cryostat set to 7 °C. Then, the excitation and emission fluorescence spectra (band‐pass 3 nm) of the standard quinine sulfate (*E*_345_ = 0.0016) and of the plasma membrane vesicle preparation (70 μg membrane protein·mL^−1^ in 20 mm potassium phosphate, pH 7.2, 100 mm NaCl) were measured. Next, 2 μL of the 0.14 mm *n*‐(9‐anthroyloxy) fatty acid stock solution were added to 1400 μL of plasma membrane vesicle suspension (probe : phospholipid molar ratio ~ 1 : 200–1 : 400), mixed by vortexing and incubated in the dark for 12 h at 4 °C. The next morning the fluorescence cuvette (Hellma, Müllheim, Germany) was filled with 500 μL of the fluorescent plasma membrane vesicle suspension and excitation and emission fluorescence spectra (band‐pass 3 nm) were recorded at 10 °C.

The temperature of the solution was measured with an accuracy of ±0.02 °C using a calibrated thermistor (type YSI 44006; Knauer, Berlin, Germany) glued with UHU plus into a glass capillary that extended through the stopper of the cell into the solution directly above the light path [28]. The thermistor formed one branch in the circuit of a precision temperature bridge (Knauer).

To monitor the temperature dependence of the anisotropy of the *n*‐(9‐anthroyloxy) fatty acid in the vesicles, the Glan Thompson prisms were installed in the optical workbench of the fluorimeter. Starting at 10 °C, the temperature in the cuvette was increased at a rate of 12 °C·h^−1^ up to 52 °C. Anisotropy was continuously measured alternatingly at the excitation wavelengths of 319, 333, 347, 367, and 381 nm, that is, the maxima of the excitation spectrum of the *n*‐(9‐anthroyloxy) fatty acid. To minimize light scatter, fluorescence emission was collected through a KV 418 cutoff filter (Schott, Mainz, Germany) at 440 nm (band‐pass 3 nm).

The fluorescence emission anisotropy was determined as follows:

$$r=\left(R_{v}–R_{h}\;\right)/\left(R_{v}+2R_{h}\right)$$

whereby *R*_v_ = *I*_vv_/*I*_vh_ represents the ratio of vertically to horizontally polarized emission light for vertically polarized excitation light, and *R*_h_ = *I*_hv_/*I*_hh_ is the calibration factor that corrects for the polarization of the instrument. All measurements with AuxB1 and B30 membrane preparations were performed with each probe in triplicate.

In case of the measurements with unilamellar DPPC vesicles in 0.1 mm potassium phosphate, pH 7, two μL of the 0.14 mm *n*‐(9‐anthroyloxy) fatty acid solution were added to 1400 μL of 70 μg DPPC·mL^−1^ buffer. After the vesicles had been incubated in the dark for 2 h at 47 °C above the gel to liquid–crystalline transition, fluorescence excitation, and emission spectra were recorded at 37 °C. Thereafter, the anisotropy was measured at 37 °C in the range of 250–400 nm excitation wavelength (band‐pass 3 nm) and fluorescence emission was collected through a KV 418 cutoff filter (Schott) at 440 nm (band‐pass 3 nm) as described above for CHO plasma membranes.

### Data analysis

The fluorescence anisotropy values of the *n*‐(9‐anthroyloxy) fatty acids were interpreted according to the evaluation procedure of Vincent *et al*. [21] based on the formalism developed by Weber [29] and Shinitzky *et al*. [30]. The steady‐state fluorescence anisotropy is determined by the statistic photon selection, the angle *α* between the absorption and emission transition dipole moments, both of which sum up as the absolute anisotropy *r*_O_ and the movement of the fluorophore. In the case of planar aromatic molecules like anthracene, the absorption and emission oscillators are coplanar. Steady‐state measurements yield an average rate of the rotation of the fluorophore determined by *r*_O_ and the two modes of rotation with respect to the absorption oscillator: *R*_op_ is an out‐of‐plane rate of rotation, that is, the rotation around the ester bond at C9 of the anthracene ring. *R*_ip_ is the in‐plane rate of rotation, that is, the acyl chain segmental reorientation motion [31]. If the rotational rate is slow so that only small rotations take place between excitation and emission, the anisotropy *r* of the fluorescent rotating plates is approximately determined by the relation:

(1) $$r_{o}/r=1+6\;\tau_{F}\left[\left(R_{\mathrm{ip}}\left(2\;\cos^{2}\alpha –1\right)+R_{\mathrm{op}}\;\cos^{2}\alpha \right)/\left(3\;\cos^{2}\alpha –1\right)\right],$$

where τ_*F*_ is the excited‐state lifetime. The variation of [(*r*_o_/*r*) – 1]/τ_*F*_ with the excitation wavelength indicates the anisotropic motion of the fluorophore [30]. If we substitute cos^2^ *α* by its expression in function of *r*_o_,

$$\cos^{2}\alpha =\left(5\;r_{o}–1\right)/3$$

Eqn (1) is written as the linear relationship [21].

(2) $$Y=R_{\mathrm{ip}}\;X+R_{\mathrm{op}}$$

with

$$Y=\left(5\;r_{o}/2\;\tau_{F}\right)\;\left(\left[\left(r_{o}/r\right)–1\right]/\left(5\;r_{o}+1\right)\right)$$

and

$$X=\left(10\;r_{o}–1\right)/\left(5\;r_{o}+1\right)$$

*Y* and *X* were calculated from the experimental *r*‐values of fluorescence emission anisotropy at the excitation wavelengths of 319, 333, 347, 367, and 381 nm. The *Y* = *f*(*X*) plots were evaluated by curve fitting and weighted linear regression. The mean excited‐state lifetime values τ_*F*_ and the zero‐time anisotropy values *r*_o_ for 2‐AS, 7‐AS, 9‐AS, 12‐AS, and 16‐AP in the temperature range of 10–50 °C were interpolated from published decay time measurements of the probes in DPPC vesicles at 21, 37, and 47 °C [21].

## Results

**Fig. 1:**
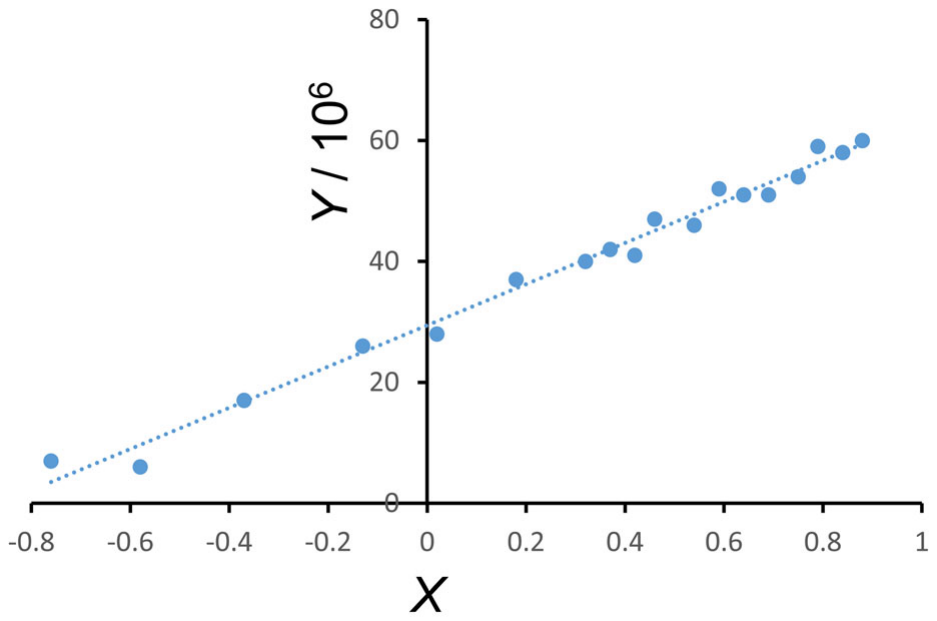
Plot of *Y* = *f(X)* for 2‐AS in DPPC vesicles at 37 °C deduced from three independent vesicle preparations. *Y* and *X* parameters are described in Material and methods in the section ‘Data analysis’ [Eqns (1) and (2)].

Vincent and colleagues [21] have characterized the fluorescence anisotropy of *n*‐(9‐anthroyloxy) fatty acids in DPPC vesicles by time‐resolved measurements. Taking this data as a reference to test the performance of our experimental setup, we measured the fluorescence anisotropy of 2‐AS, 9‐AS, and 16‐AP at 37 °C in unilamellar DPPC vesicles. Anisotropy was determined within the range of 250 to 400 nm excitation wavelength. As shown in Fig. 1 for 2‐AS, the plots of *Y* = *f(X)* (Eqn 2) were linear, suggesting that the dataset can be interpreted in terms of the formalism given in Eqn (1). Table 1 lists the computed out‐of‐plane (*R*_op_) and in‐plane (*R*_ip_) rotational rates. *R*_op_ and *R*_ip_ were almost identical for each one of the three probes. This finding is in line with the behavior of the probes in isotropic media [21].

**Table 1:** Computed out‐of‐plane (*R*_op_) and in‐plane (*R*_ip_) rotational rates^a^ and corresponding rotational correlation times (ø)^b^ of *n*‐(9‐anthroyloxy) fatty acids in DPPC vesicles at 37 °C.

Probe	*R*_op_ [MHz]	*R*_ip_ [MHz]	ø_op_ [ns]	ø_ip_ [ns]	ø [ns]^c^
2‐AS	29.1 ± 0.9	31.2 ± 1.1	5.7	5.3	5.7
9‐AS	31.5 ± 1.5	30.8 ± 1.2	5.3	5.4	4.0
16‐AP	40.6 ± 1.2	39.5 ± 1.8	4.1	4.2	1.5

^a^ Values indicate the mean and 95% confidence intervals of the linear regression of the *Y* = *f(X)* plots deduced from three independent vesicle preparations.

^b^ Calculated as ø_op_ = 1/(6 *R*_op_) and ø_ip_ = 1/(6 *R*_ip_) according to Yguerabide *et al*. [65].

^c^ ø values were taken from Fig. 10 in Vincent *et al*. [21] who had measured the anisotropy at the excitation wavelength of 316 nm (10‐nm band‐pass) where *r*_o_ is close to 0.1. Hence, the anisotropy is predominantly determined by the out‐of‐plane rotation *R*_op_ (see Eqn 2).

**Fig. 2:**
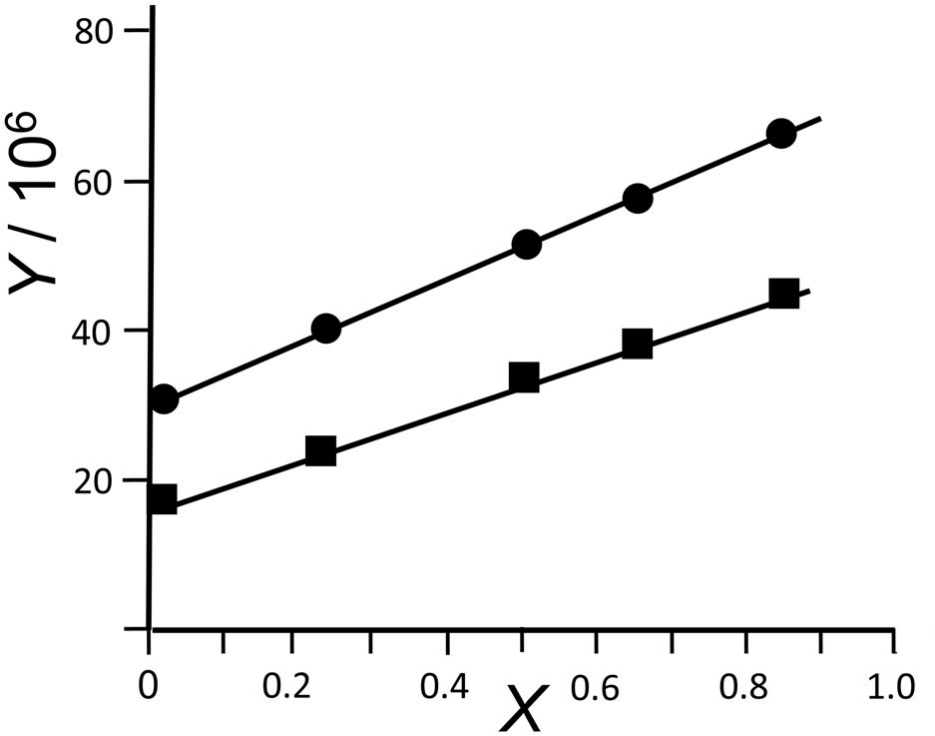
Plot of *Y* = *f(X)* for 2‐AS in AuxB1 (solid circles) and CH^R^B30 (solid squares) plasma membranes at 20 °C (*n* = 3 technical replicates). *Y* and *X* parameters are described in Material and methods in the section ‘Data analysis’ [Eqns (1) and (2)].

Based on these findings on DPPC vesicles that the plots of *Y* = *f(X)* were linear and that our steady‐state measurements generally concur with the time‐resolved fluorescence anisotropy measurements, we applied the formalism of Weber [29] and Shinitzky *et al*. [30] to our steady‐state measurements of the anisotropy of plasma membranes of drug‐sensitive AuxB1 and multidrug‐resistant CH^R^B30 CHO cells. The temperature dependence of fluorescence anisotropy of 2‐AS, 7‐AS, 9‐AS, 12‐AS, and 16‐AP in the range of 10 to 52 °C was recorded with the same hardware and the same protocol within the study period of 2 years. During each experiment, the anisotropy at the excitation wavelengths of 319, 333, 347, 367, and 381 nm was measured a total of 98 to 180 times (median 130 times) corresponding to 23–43 anisotropy data points within a temperature interval of 10 °C. Regression analysis revealed linear *Y* = *f(X)* plots (see Fig. 2 for 2‐AS at 20 °C as an example) confirming that Eqn (2) could be applied to compute out‐of‐plane and in‐plane rotational rates from our heating curves. Within experimental error, each of the *n*‐(9‐anthroyloxy) fatty acids showed matching quantum yields, fluorescence emission spectra, and uptake kinetics in AuxB1 and CH^R^B30 vesicles indicating that the probes partitioned with the same yield into the plasma membranes of drug‐sensitive AuxB1 and multidrug‐resistant CH^R^B30 CHO cells. Likewise, 9,10‐dimethylanthracene and methyl 9‐anthracene carboxylic acid, which can be considered as surrogate compounds of the fluorescence label in the *n*‐(9‐anthroyloxy) fatty acid probes, showed matching partitioning and spectrofluorometric properties in AuxB1 and CH^R^B30 plasma membrane vesicles. Conversely, when we exposed the plasma membranes to the fluorescent anthracycline daunomycin, a known substrate of P‐glycoprotein [32], 1.4‐fold more daunomycin partitioned into B30 than into AuxB1 vesicles.

**Fig. 3:**
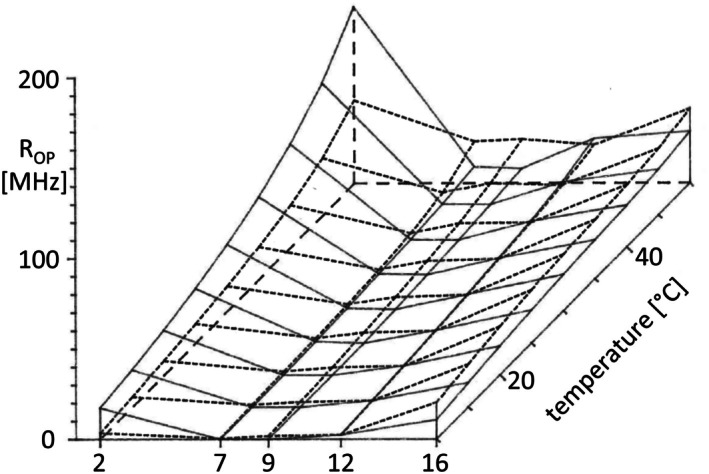
Transversal gradient of the rotation correlation coefficient *R*_op_, the out‐of‐plane rotation around the ester bond at C9 of the anthracene ring of *n*‐(9‐anthroyloxy) fatty acid probes (*n* = 2, 7, 9, 12, 16) embedded into plasma membranes of the drug‐sensitive CHO cell line AuxB1 (solid line) and of its highly multidrug‐resistant derivative CH^R^B30 (dashed line) within the range of 10–50 °C. Heating curves were recorded for each probe and each cell line in triplicate.

**Fig. 4:**
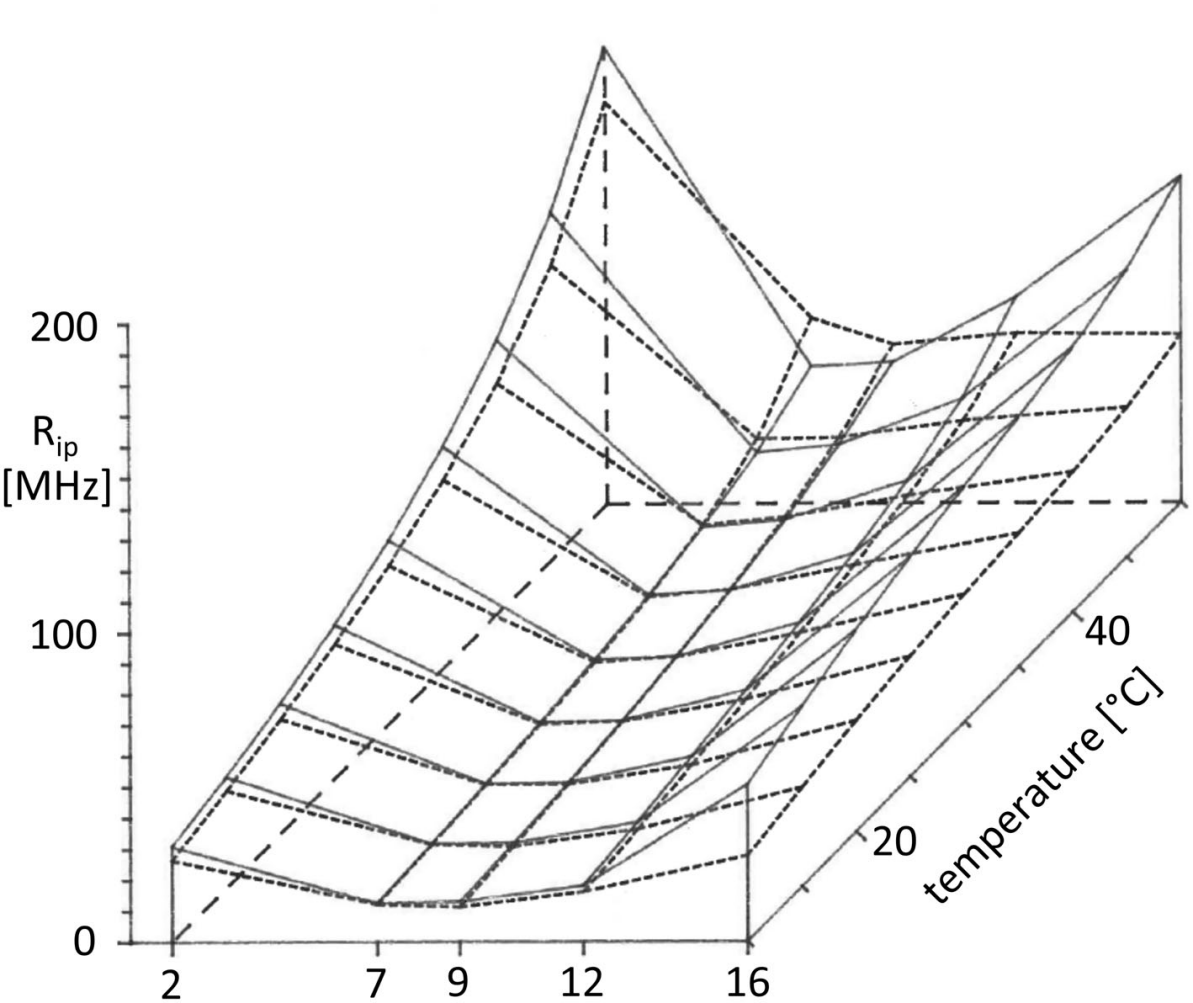
Transversal gradient of the rotation correlation coefficient *R*_ip_, the in‐plane acyl chain segmental reorientation motion of *n*‐(9‐anthroyloxy) fatty acid probes (*n* = 2, 7, 9, 12, 16) embedded into plasma membranes of the drug‐sensitive CHO cell line AuxB1 (solid line) and of its highly multidrug‐resistant derivative CHRB30 (dashed line) within the range of 10–50 °C. Heating curves were recorded for each probe and each cell line in triplicate.

The three‐dimensional plots in Figs 3, 4 show the computed *R*_op_ and *R*_ip_ values of the *n*‐(9‐anthroyloxy) fatty acids from 10 to 50 °C in AuxB1 and CH^R^B30 plasma membranes. The continuous change of *R*_op_ and *R*_ip_ with temperature was derived for each probe from the whole dataset of three heat curves. The change of *R*_op_ and *R*_ip_ along the transversal gradient of the position in the aliphatic hydrocarbon chain that is esterified with the fluorophore was interpolated from the heat curves in steps of 5 °C.

When we compare the *R*_op_ and *R*_ip_ profiles in AuxB1 and CH^R^B30 membranes (Figs 3 and 4), similarities in the overall shape, but also distinct differences, are apparent. The rotational rates continuously increased with temperature and consistently had maxima for 2‐AS and 16‐AP and low or very low values for 7‐AS, 9‐AS, and 12‐AS across the entire temperature range. *R*_ip_ was always higher than *R*_op_ for concurring experimental conditions of probe, cell line, and temperature.

The transversal gradients of *R*_op_ and *R*_ip_ differed between CH^R^B30 and AuxB1 plasma membranes. At corresponding positions of probe and temperature, the rotational rates varied up to twofold between the two CHO lines. The *R*_ip_ profile was almost matching between AuxB1 and CH^R^B30 for 2‐AS, 7‐AS, 9‐AS, and 12‐AS, but *R*_ip_ values of 16‐AP were about twofold higher in AuxB1 throughout the entire temperature range. Conversely, the *R*_op_ profiles were distinct in AuxB1 and CH^R^B30 membranes. The transversal gradient was smoother in CH^R^B30 than in AuxB1 membranes. The *R*_op_ values in CH^R^B30 were lower for 2‐AS, matched with AuxB1 for 12‐AS, and were higher for 7‐AS, 9‐AS, and 16‐AP.

**Fig. 5:**
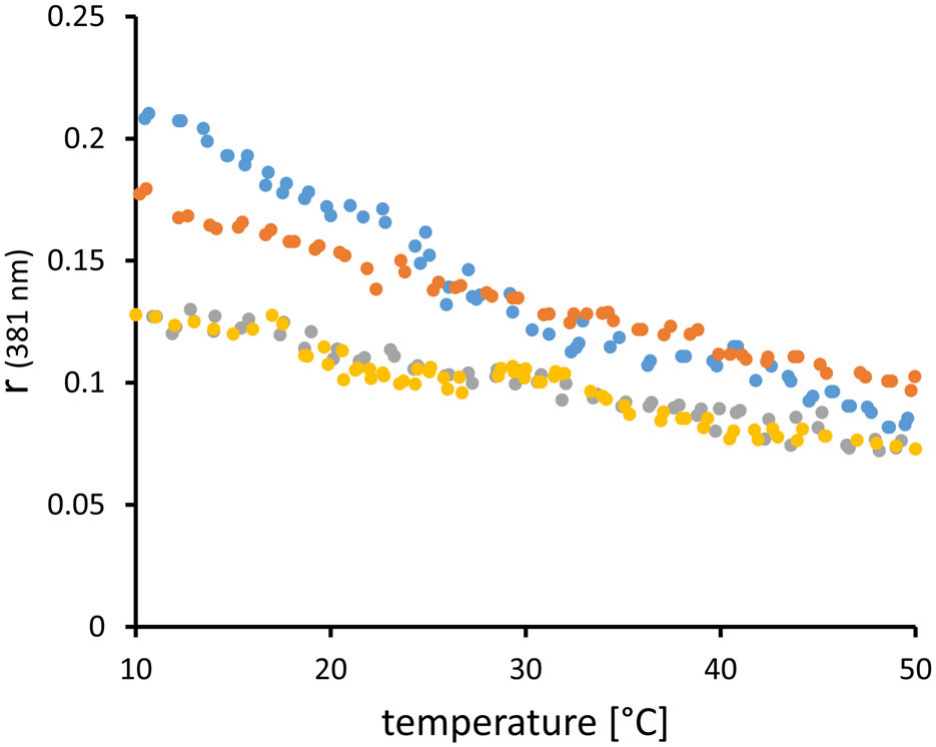
Single recordings of the fluorescence anisotropy of 2‐AS at 381 nm embedded in AuxB1 (gray dots, yellow dots) and CH^R^B30 (orange dots, blue dots) plasma membranes in the absence (gray dots, orange dots) and presence (yellow dots, blue dots) of 10 μm verapamil within the range of 10–50 °C. The dots represent the individual measurements at 381 nm during the recording of the heating curves.

**Fig. 6:**
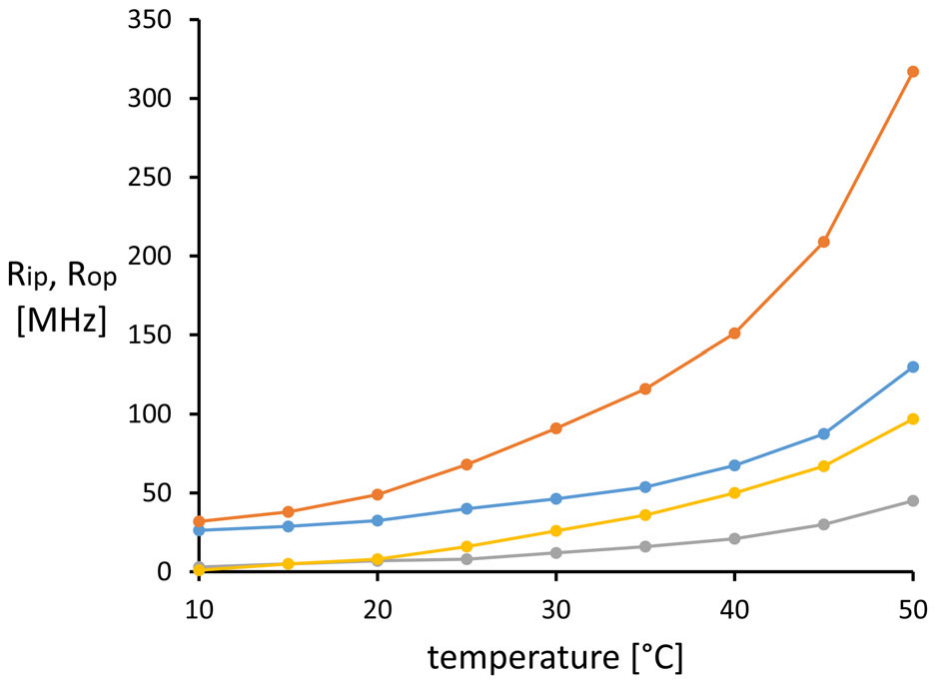
Rotation correlation coefficients *R*_op_ (gray and yellow lines) and *R*_ip_ (blue and orange lines) of the probe 2‐AS embedded in CH^R^B30 plasma membranes in the absence (gray and blue lines) and presence (yellow and orange lines) of 10 μm verapamil within the range of 10–50 °C (*n* = 2 technical replicates).

The heating curves were recorded in phosphate buffer at pH 7.2 in the absence of ATP or any substrate or inhibitor of P‐glycoprotein. Hence, P‐glycoprotein was monitored in the apo state that according to the recently published cryo‐EM data is characterized by ‘an inward‐facing closed state with closely spaced NBDs and a closed TMD pathway central’ (citation from the Results section of ref. [4] on the apo‐ABCB1 conformation). To address this issue whether the conformational state of P‐glycoprotein may modulate the *R*_op_ and *R*_ip_ profiles of the plasma membranes, we repeated the heating curves with 2‐AS in the absence and presence of 10 μm verapamil, the historically first detected inhibitor of P‐glycoprotein [33]. The temperature profile of the fluorescence anisotropy of 2‐AS remained invariant in AuxB1 membranes but changed in CH^R^B30 membranes upon exposure to verapamil (Fig. 5). Both *R*_ip_ and *R*_op_ values monotonously increased by about 1.2‐fold at 10 °C up to 2.4‐fold at temperatures above 40 °C (Fig. 6). These data imply that the verapamil‐induced change of the conformational state of P‐glycoprotein increased the mobility of 2‐AS as a measure of the fluidity of the CH^R^B30 membrane.

## Discussion

The steady‐state fluorescence anisotropy measurements of *n*‐(9‐anthroyloxy) fatty acid probes in apo‐P‐glycoprotein overexpressing membranes revealed that the overexpression of hamster P‐glycoprotein in CHO cells smoothened the transversal gradient of the rate of rotation around the ester bond at C9 of the anthracene ring of the *n*‐(9‐anthroyloxy) fatty acid probes, whereas the acyl chain segmental reorientation motion of the probe was only affected in the hydrophobic midst of the plasma membrane. Multidrug resistance against chemotherapeutic agents and its association with the overexpression of the multidrug‐transporter P‐glycoprotein in the plasma membrane have been discovered in the CHO cell lines [1, 14, 34] that are the subject of the current study. The plasma membranes used for the fluorescence measurements were purified from CHO cells according to the same protocol that visualized for the first time P‐glycoprotein in the Coomassie stain of vesicles from drug‐resistant cells [1]. With the exception of the high abundance of P‐glycoprotein in the plasma membrane vesicles of drug‐resistant cells, the drug‐sensitive ancestor AuxB1 and its colchicine‐resistant derivatives showed the same polypeptide composition in the SDS/PAGE of membrane proteins [1] and, within experimental error, the same phospholipid and cholesterol contents, the same fatty acid and phospholipid composition, and the same fatty acid composition of phospholipid classes [18]. Hence, we can assign the differential mobility of the *n*‐(9‐anthroyloxy) probes in AuxB1 and CH^R^B30 membranes to the differential amounts of P‐glycoprotein as the only detected distinction. The overexpression of P‐glycoprotein is associated with a change in membrane ultrastructure at the protoplasmic face [15] that should influence the mobility of the probes. Freeze‐fracture electron microscopy demonstrated that the protoplasmic face of CH^R^B30 plasma membranes was characterized by a higher density of intramembrane particles 6–9 nm in diameter (~ 2400·μm^2^) than the protoplasmic face of AuxB1 membranes (~ 200·μm^2^) [15].

In CH^R^B30 cells, P‐glycoprotein is localized in intermediate‐density membrane microdomains different from caveolar domains or classical lipid rafts [35]. P‐glycoprotein thereby directly interacts with the lipid bilayer [36, 37] and binds cholesterol [38] and lipids [39]. The lipid environment modulates the function of P‐glycoprotein such as ATP hydrolysis, drug binding, and drug transport (reviewed by Hegedüs *et al*. [12] and Sharom [13]). Moreover, P‐glycoprotein incorporates lipids in its hydrophobic cavity, and its conformational changes in the cytoplasmic halves of all transmembrane domains are stabilized by dynamic lipid contacts [4]. On the other hand, the temperature profile of the rotational mobility of *n*‐(9‐anthroyloxy) fatty acids is distinct in AuxB1 and CH^R^B30 membranes, indicating that the overexpression of P‐glycoprotein modulates the global transversal gradients of mobility and microviscosity of the plasma membrane.

We selected the *n*‐(9‐anthroyloxy) fatty acids as the appropriate set of reporter molecules for parameters related to membrane penetration depth [40, 41, 42, 43, 44, 45]. In model lipid membranes, the distance of the anthroyloxy group from the bilayer center is almost linearly related to the number of carbon atoms between the anthroyloxy and carboxyl groups [41]. Hence, the fluorescence anisotropy for a particular *n*‐AS probe is a measure of a depth‐dependent property of the membrane. We measured fluorescence emission at the emission maximum where the fluorescence decay is monoexponential [46, 47] implying that the dependence on the anisotropy from excitation wavelength reflects the local rotational dynamics of the 9‐anthroyloxy group [21, 31]. With the chosen probe‐to‐lipid ratio of 1 : 200–1 : 400, the structure of the membrane is not perturbed by the fluorescent fatty acid [43]. Thus, the calculated *R*_ip_ and *R*_op_ rotational rates should reflect the real‐world local environment at different depths within the AuxB1 and CH^R^B30 plasma membranes that solely differ by a factor of about 20 in their content of P‐glycoprotein [14, 15]. P‐glycoprotein of CH^R^B30 cells is known to function as a phospholipid flippase [48, 49] and as an efflux pump for a wide range of amphiphilic and hydrophobic substrates [50, 51]. However, unlike the phospholipids, the anthroyloxy fatty acids are probably not substrates of hamster P‐glycoprotein. At the standardized concentration of 0.2 μm, the probes equilibrated within 80–120 min in AuxB1 and CH^R^B30 membrane vesicles and then showed identical spectrofluorometric properties. Conversely, established substrates or inhibitors of P‐glycoprotein, such as daunomycin, vinblastine, or verapamil equilibrated within a few seconds, were embedded in higher amounts into CH^R^B30 than into AuxB1 plasma membrane vesicles and showed high‐affinity binding to P‐glycoprotein ([51, 52], own unpublished data). Thus, the anisotropy profiles of the anthroyloxy fatty acids in CH^R^B30 membranes could have been influenced by direct and/or indirect interactions with P‐glycoprotein (for possible binding interactions see e.g., refs. [4, 6]).

Applications of the anthroyloxy fatty acid probes to real biological membranes are scarce. Most studies analyzed the probes in isotropic solvents, lipid vesicles, or single protein/metabolites incorporated into vesicles. Published studies on the fluorescence anisotropy of the probes in biological membranes [53, 54, 55, 56] were confined to a single excitation wavelength and hence did not allow any calculation of rotational rates of the fluorophore. Thus, more than 50 years after this series of probes had been designed for membrane studies [57], the probes have now been used for the first time in a biological membrane to cover a representative temperature range of 40 °C from the broad phase of lipid melting up to a fluid lipid layer when the proteins start to unfold [58].

The measurements on the plasma membrane vesicles were performed in aqueous phosphate buffer in the absence of ATP, substrates, or inhibitors of P‐glycoprotein. Thus, P‐glycoprotein was in the physiologically inactive apo conformational state [4]. The in‐plane and out‐of‐plane rotations of the fluorophore show similar profiles in their dependence on temperature and membrane penetration depth (Figs 3, 4). In contrast to phospholipid vesicles that show linear gradients of rotational mobility along the transversal path of the bilayer [21], the rates of rotation in the CHO membranes close to the lipid/aquatic interface and in the hydrophobic core of the bilayer are high and low in between at carbons 7, 9, and 12 of the fatty acid. This finding that the mobility of molecules has a minimum in the area of intermediate polarity is typical for biological membranes and has been interpreted as ‘the microviscosity barrier of the bilayer’ [59]. The in‐plane rotations of the acyl chain of the probes are not affected by P‐glycoprotein at the positions 2, 7, 9, and 12, but at the hydrophobic terminus. P‐glycoprotein apparently does not influence the motion of the acyl chain in the more polar regions of the bilayer, but restricts the mobility of its lipid environment in the apolar center. An opposite, but even stronger impact of the overexpression of P‐glycoprotein was seen for the transversal gradient of the rotation of the probe around the ester bond at C9 of the anthracene ring. The transversal profile of out‐of‐plane rotations is smoothened. Thus, hamster apo‐P‐glycoprotein modulates the ‘fluidity gradient’ of the CH^R^B30 plasma membrane. Particularly at higher temperature above 30 °C, the mobility has still its minimum at positions 7 and 9, but the gain of mobility is substantial in comparison to AuxB1 membranes. It is tempting to assume that this modulation of the *R*_op_ profile may facilitate the partitioning of hydrophobic drugs into the membrane and thereby increases the speed of the multidrug‐transporter to pump the drug out of the cell. For example, we have compared the release of drug from AuxB1 and CH^R^B30 cells with pulsed quench‐flow kinetic analyses ([60, 61, 62], unpublished data). Within 15 s, CH^R^B30 cells released more than 90% of the load of daunomycin and 25% of equal amounts of vinblastine. The drug‐sensitive AuxB1 cells expelled 10% of daunomycin and 13% of vinblastine within 15 s. Thus, we would like to conclude that P‐glycoprotein modulates its lipid environment to increase the efficacy of the binding and transport of amphiphilic and hydrophobic substrates.

We measured the fluidity gradient of highly purified CHO plasma membranes to generate high‐quality data. However, this approach has the inevitable disadvantage that we investigated hamster P‐glycoprotein in the apo state [4], which of course is not the physiological conformation of P‐glycoprotein in the living cell assessed in our quench‐flow experiments. Motivated by the valuable reviewers' comments to the first version of the manuscript, we have repeated the heating curves with 2‐AS in the absence and presence of verapamil to which CHO B30 cells are hypersensitive [63, 64]. The inhibitor verapamil binds with high affinity to hamster P‐glycoprotein [51]. Exposure to verapamil did not affect the *R*_ip_ and *R*_op_ temperature profiles of AuxB1 membranes but increased the in‐plane and out‐of‐plane rotations of the probe in CH^R^B30 membranes over the whole temperature range of 10–50 °C (Fig. 6). Since AuxB1 and CH^R^B30 membranes differ in the amount of P‐glycoprotein but otherwise match in their lipid [18] and polypeptide composition [1], the measured increased mobility of 2‐AS in fluidity of the CH^R^B30 membranes may be ascribed to the higher density of potentially membrane‐disordering intramembrane particles [15] or to a verapamil‐induced change of the conformational profile of P‐glycoprotein from the apo state to the complex with substrate/inhibitor in the absence of ATP/Mg^2+^ [4]. Based on these preliminary findings and our current knowledge of four distinct conformations of P‐glycoprotein [4], this issue remains to be evaluated more extensively whether the conformational spectrum of P‐glycoprotein modulates the fluidity of the plasma membrane in multidrug‐resistant P‐glycoprotein‐overexpressing cells.

## Author contributions

JRR and BT designed the study. RB and BT performed the spectrofluorometric experiments and evaluated the anisotropy data. JRR provided the CHO cell lines and isolated the plasma membranes. BT wrote the manuscript.

## Conflict of interest

The authors declare no conflict of interest.

## Peer review

The peer review history for this article is available at https://www.webofscience.com/api/gateway/wos/peer‐review/10.1002/1873‐3468.70083.

## Data Availability

The data are incorporated into the body of the manuscript.
